# Developing the PATH-GP (Prevention and Testing for HIV in General Practice) intervention: a person-based approach intervention development study to increase HIV testing and PrEP access

**DOI:** 10.3399/BJGP.2025.0151

**Published:** 2025-12-02

**Authors:** Anne Scott, Hannah Family, Jeremy Horwood, John Saunders, Ann Sullivan, Jo Burgin, Lindsey Harryman, Sarah Stockwell, Joanna Copping, Paul Sheehan, John Macleod, Sarah Dawson, Joanna May Kesten, Sarah Denford

**Affiliations:** 1 National Institute for Health and Care Research Applied Research Collaboration West at University Hospitals Bristol and Weston NHS Foundation Trust, Bristol, UK; 2 Population Health Sciences, Bristol Medical School, University of Bristol, Bristol, UK; 3 National Institute for Health and Care Research, Health Protection Research Unit in Behavioural Science and Evaluation, Bristol, UK; 4 Centre for Academic Primary Care, Bristol Medical School, University of Bristol, Bristol, UK; 5 Blood Safety, Hepatitis, STI and HIV Division, UK Health Security Agency, London, UK; 6 Chelsea and Westminster Hospital NHS Foundation Trust, London, UK; 7 Unity Sexual Health, University Hospitals Bristol and Weston NHS Foundation Trust, Bristol, UK; 8 Cardiff and Vale University Health Board, Cardiff, Wales, UK; 9 Communities and Public Health, Bristol City Council, Bristol, UK; 10 Public Health and Prevention, Bath and North East Somerset Council, Bath, UK

**Keywords:** behaviour change interventions, family practice, general practice, HIV testing, pre-exposure prophylaxis, qualitative research

## Abstract

**Background:**

Testing for HIV, linkage to treatment, and access to pre-exposure prophylaxis (PrEP) (medication that reduces the risk of acquiring HIV) is essential for early HIV diagnosis, treatment, and prevention. General practice could play a key role in maximising HIV testing opportunities and supporting access to PrEP.

**Aim:**

To develop an intervention for general practice to increase HIV testing and facilitate access to PrEP.

**Design and setting:**

This was a person-based approach (PBA) intervention development study using the capability, opportunity, motivation, behaviour model in South West England.

**Method:**

A scoping review and semi-structured interviews with healthcare professionals (HCPs) and local organisation representatives with an interest in HIV prevention/health care were conducted to understand the challenges and find potential solutions to increase HIV testing and facilitate access to PrEP in general practice. Intervention development used focus groups with HCPs and the public. Purposive sampling ensured diversity of practices and participants. Data were analysed using the PBA table of planning and the collaborative and intensive pragmatic qualitative approach.

**Results:**

Barriers identified included lack of clinician knowledge of HIV and PrEP, concern about stretched resources, and a lack of systematic testing methods. Proposed strategies included simpler testing approaches to normalise testing and reduce HIV stigma. The intervention developed consists of: education, a prompt to test, simplified and standardised testing, PrEP signposting processes, patient information, and practice champions.

**Conclusion:**

Research is needed to explore the feasibility and the effectiveness of this multicomponent intervention to increase testing and access to PrEP within general practice. Funding barriers also need to be addressed.

## How this fits in

General practice could play a key role in maximising HIV testing opportunities and supporting access to pre-exposure prophylaxis (PrEP). Missed testing opportunities continue to contribute to late diagnoses, poorer health outcomes, and higher treatment costs. Although patient acceptability for HIV testing is high, testing rates remain low and variable and access to PrEP is often limited. This study developed an intervention combining education, training, and systematic testing approaches to address barriers and improve HIV prevention in general practice.

## Introduction

The UNAIDS global goal to end new HIV infections by 2030,^1^ requires coordinated efforts to improve HIV prevention, detection, and treatment. In the UK, this is a high priority for NHS England and the Department of Health and Social Care as outlined in the HIV Action Plan^2^ for England and National Institute for Health and Care Excellence (NICE) guidance.^3,4^

In 2023, 4700 people in England were estimated to be living with undiagnosed HIV^5^ and 923 of people first diagnosed in England were diagnosed late.^5,6^ Reducing late HIV diagnosis (defined as a CD4 count of <350 cells within 91 days of diagnosis) and missed opportunities for HIV testing are central to the elimination of HIV transmission.^2–7^ Late HIV diagnosis is associated with increased risk of hospital admissions, reduced life expectancy, increased mortality, and greater treatment and secondary care costs.^8–10^ Conversely, HIV antiretroviral therapy increases life expectancy in line with the general population^11^ and those on effective treatment with an undetectable viral load cannot pass HIV on.^12^ Routine HIV testing in high HIV prevalence areas is cost-effective in the medium term.^13^ Studies demonstrate high levels of patient acceptance and acceptability of HIV testing through general practice.^14–16^ General practice could therefore play a central role in early HIV diagnoses. Despite this, HIV testing rates in general practice are low and variable^17,18^ and patients often present with HIV-related indicator conditions several times before being diagnosed with HIV^19,20^ and primary or early-stage HIV infection is frequently missed.^21^

In areas of high prevalence (>2 per 1000 people aged 15–59, for example, Bristol 2.37 per 1000 people)^22^ and extremely high prevalence (>5 per 1000 people aged 15–59) guidance from NICE and the British HIV Association (BHIVA)^3,23,24^ recommends offering testing to all new registrants, to patients presenting with indicator conditions and to patients undergoing blood tests for any other reason. Offering testing in high HIV prevalence areas according to this guidance is cost-effective in the medium term.^13^ In addition, in areas of extremely high prevalence HIV testing should be considered opportunistically at each consultation. In low-prevalence areas, guidelines recommend testing in response to indicator conditions and testing people who may be exposed to HIV.^24^ HIV testing is important in all areas^25^ and requires adequate resources and commissioning^26^ to pay for staff training and increased testing. Evidence suggests that testing outside specialist genitourinary medicine (GUM)/sexual health services (SHS) does not fully adhere to these guidelines.^27,28^

Increasing general practice HIV testing in line with the recommendations above also provides opportunities to identify patients eligible for pre-exposure prophylaxis (PrEP)^29^ a biomedical intervention that reduces the risk of getting HIV from sex by 99%.^7,29,30^ PrEP should be offered to people who may benefit from protection against HIV using criteria in the BHIVA/British Association for Sexual Health and HIV (BASHH) guidelines.^4,31^ Increasing access to PrEP, is an important, potentially cost-effective^4,32^ means of preventing new HIV infections. PrEP is currently only available via NHS specialist level 3 GUM services, which leads to inequalities in access among some patient groups who use SHS less^6^ and calls have been made for interventions to raise awareness of PrEP in women, Black and ethnic minority and transgender populations^33^ who were underrepresented in the PrEP Impact trial.^34^

General practice could play a crucial role in increasing PrEP access by informing patients about PrEP, and referring them to specialist services.^4,35^ There is currently a dearth of evidence on whether general practice healthcare professionals (HCPs) in England are supporting access to PrEP^36^ and a lack of interventions to support increased awareness of PrEP and signposting to PrEP services in general practice.

Education interventions for HCPs can increase HCP awareness, confidence, and consideration of HIV testing but may not address opportunity and motivation barriers to testing,^37^ and the impact on testing varies.^18,38–41^ Multicomponent, complex interventions, may enhance the effects of educational interventions on HIV testing behaviours.^42,43^

To increase HIV testing in high- and low-prevalence areas there is a need to utilise a validated approach to intervention development, incorporating behaviour change theory and interest holder involvement to maximise acceptability and effectiveness.^44^ This article presents the development of a complex, theory-based general practice intervention to increase HIV testing and support access to PrEP.

## Method

**Figure 1.**
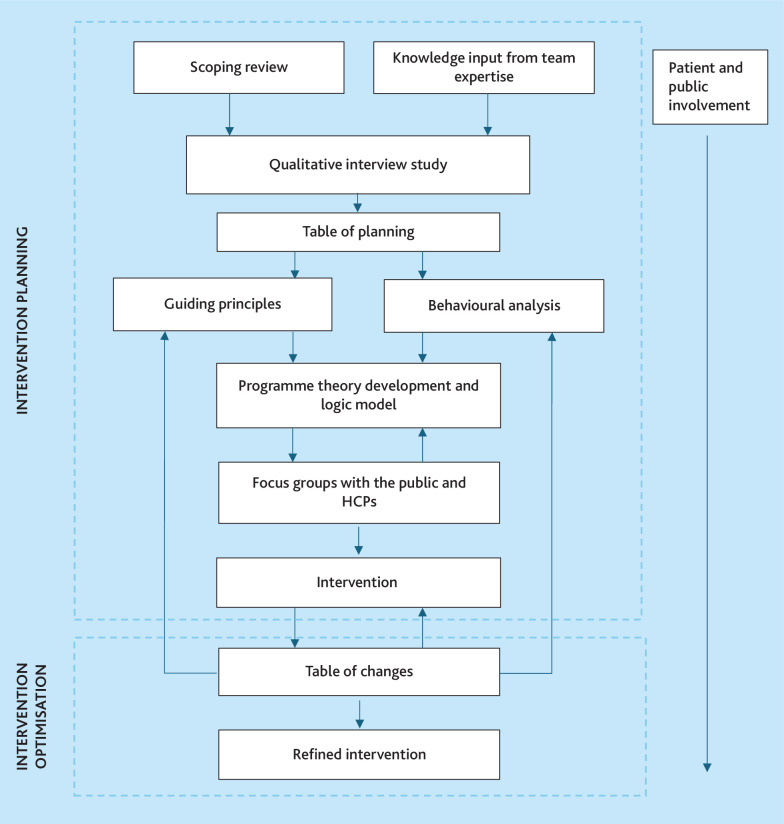
Person-based approach (PBA) study design overview. HCP = healthcare professional.

The study, conducted between October 2022 and August 2024, was guided by the person-based approach (PBA)^44^ and the collaborative and intensive pragmatic qualitative (CLIP-Q) approach (Figure 1).^45^ PBA aims to develop interventions tailored to the views, needs, and experiences of the individuals who use them. This iterative approach to planning and optimising ensures that the intervention is relevant, engaging, and easy to use.^44^ The CLIP-Q approach complemented the PBA by ensuring that all interested parties were involved from an early stage so that key questions were identified and incorporated into data collection. Using CLIP-Q enabled the rapid collection and analysis of data, which is particularly suitable for the exploration of highly focused topics.^45^

### Evidence and theory

A scoping review of systematic reviews to understand the barriers and facilitators to increasing HIV testing and access to PrEP in general practice was conducted. It was undertaken in accordance with the Joanna Briggs Institute methodology for scoping reviews.^46^ Searches were performed in Ovid Medline, Ovid APA PsycINFO, Web of Science Core Collection, Ovid Embase. Additionally, we searched the following review databases: Epistmonikos, Health Evidence, Database of Promoting Health Effectiveness Reviews (DoPHER) and NIHR Health Technology Assessments. All types of study were included (see example search strategies Supplementary Box S1). Databases were searched in December 2022 (PrEP search) and February 2023 (HIV testing search). A grey literature search was also conducted, including: Google, other relevant databases, charity, organisation, and healthcare websites.

### Patient and public involvement

Three members of the public all with different backgrounds and experiences relating to HIV helped refine the study design and participant-facing materials, topic guides, data analysis, and intervention planning and optimisation. Public contributors joined the team from the NIHR Health Protection Research Unit public panel, the Common Ambition Bristol project, and in response to a call for public involvement in the project through the local sexual health service.

Meetings were held with local interest holders involved in SHS and a charity supporting individuals living with HIV to support all aspects of the study.

### Qualitative data collection

#### Setting

The setting was general practices from low and high HIV prevalence areas in South West England (specifically, Bristol, North Somerset and South Gloucestershire; Gloucestershire; and Bath and North East Somerset).

#### Qualitative interviews with staff and interest holders

Semi-structured telephone or video interviews were conducted with general practice HCPs (including GPs, practice pharmacists, practice nurses, phlebotomists) and interest holders (such as commissioners) to understand attitudes towards increasing HIV testing and access to PrEP in general practice.

#### Participants

The Clinical Research Network invited all research-active general practices in the study areas. Participating practices responded to this invitation. These practices reflected a range of HIV testing data quartiles, HIV prevalence (low versus high) using Summary Profile of Local Authority Sexual Health reports,^47^ urban and rural area, and Indices of Multiple Deprivation. We focused on ensuring diversity of HIV testing rates initially, followed by the other categories. We carried out further recruitment to ensure that rural areas were represented. Testing data was obtained from a regional laboratory, expressed as a rate of general practice population size and the distribution categorised by quartile ranges.

#### Materials and procedures

Twelve GP practices emailed their staff information about the study and interested staff contacted the research team to arrange an interview. The interview topic guide was informed by the results of the scoping review and COM-B model^48^ and was modified in response to issues raised in earlier interviews (see Supplementary Box S2). Interviews were audio-recorded with consent, professionally transcribed, and anonymised. Interview participants or their practices were reimbursed for their time according to the National Institute of Health and Care Research rates of pay.

#### Focus groups with staff and the public

Members of the public and HCPs were invited to separate online focus groups (of up to 1 h duration) to refine the guiding principles for the intervention and co-develop the intervention content. Guiding principles present the key contextual issues and design objectives required to facilitate behaviour change and were developed iteratively.

Members of the public with a range of characteristics (such as, gender, age, ethnicity) and experiences of HIV testing and/or PrEP use were recruited with the assistance of health ambassadors, public involvement, and applied health researchers and their networks (with ability to reach diverse groups). This was to ensure we included the voices of those often underrepresented in research, those more likely to experience late HIV diagnosis, and those who are less likely to attend sexual health clinics (such as people from ethnic minoritised communities).

General practice HCPs with a mix of clinical backgrounds and experiences of HIV testing and/or PrEP delivery were recruited from those who took part in the interviews or expressed an interest but had not yet participated.

#### Analysis

In line with the CLIP-Q approach we initially charted the interview responses using a framework matrix^45^ within Microsoft Excel. This concentrated on the study’s aims and areas of interest using deductive categories: knowledge of HIV and PrEP and barriers and facilitators to increasing HIV testing and PrEP access. This was supplemented with short illustrative quotes from the transcripts. This approach is particularly suitable when interviews are highly focused.^45^

Behaviour change theory was used to carry out the analysis. PBA outlines a range of approaches designed to incorporate behaviour change models/theories, evidence, and experience.^44^ We used this approach to ensure that the intervention was underpinned by appropriate behaviour change techniques. COM-B posits that a behaviour (B), for example, HIV testing, is influenced by an individual having capabilities (C) (skills and knowledge), opportunity (O), (social and physical), and motivation (M) (automatic emotion and reflective beliefs, intentions).

We used the PBA table of planning to record key findings on barriers and facilitators extracted from the scoping review and interview findings. Combining the key findings in a shared document assists in team discussions about intervention development.^49^ These were subsequently mapped to the COM-B model of behaviour change (see Supplementary Table S1 – a combination of behavioural analysis and the table of planning). This aimed to identify approaches that would enhance individuals’ capability, create opportunities for behaviour change, and provide motivation to increase HIV testing and access to PrEP. From this we developed an accompanying logic model (see Supplementary Figure S1), guiding principles for the intervention (Supplementary Table S2), and a draft intervention for discussion in focus groups.

Feedback from the focus groups were documented in a PBA table of changes. Suggested changes were coded by importance in relation to the guiding principles, number of times the comment was raised, and whether evidence from behaviour change theory suggests the change is likely to increase the chance of the behaviour being performed.

## Results

The scoping review to support access to PrEP, identified 339 publications of which 99 were excluded as duplicates, 163 were excluded based on title and abstract, and 46 were excluded based on full-text leaving 31 included.^50–80^ The scoping review for HIV testing identified 393 publications of which 121 were excluded as duplicates, 205 were excluded based on title and abstract, and 50 were excluded on full-text reading. A further 7 papers were found through additional searches. A total of 24 were included.^16,17,39,40,81–100^

The scoping review and interview findings are presented in Supplementary Table S1. Most systematic reviews were mixed methods including qualitative and quantitative studies.

In total, 25 HCPs (20 GPs, four practice nurses, and one pharmacist) from 12 general practices and four interest holders (representing SHS, an HIV charity, and commissioning organisations) participated in interviews lasting an average of 34 mins. Tables 1 and 2 provide demographic information about general practices and participants, respectively (to preserve anonymity interest holder demographics are not presented). Interview findings are framed using the COM-B model.

**Table 1.** Practice characteristics

General practice	HIV testing quartile group^a^	List size^b^	IMD decile^c^	Area	HIV prevalence	Staff Interviewed, *n*
1	Group 4	Medium	4	Suburban	High (2 to <5) on the boundary with low (1 to <2)	4
2	Group 2	Medium	10	Suburban	Low (1 to <2)	1
3	Group 4	Medium	5	Suburban	Low (1 to <2) on the boundary with high (2 to <5)	1
4	Group 4	Small	10	Suburban	Low (1 to <2)	1
5	Group 2	Medium	9	Suburban	Low (1 to <2) in between high prevalence areas	2
6	Group 2	Very large	5	Urban	High (2 to <5)	2
7	Group 1	Very large	10	Suburban and rural	Low (<1)	4
8	Group 4	Medium	2	Urban	High (2 to <5)	3
9	Group 1	Large	8	Urban	High (2 to <5)	1
10	Group 1	Large	10	Urban	Low (1 to <2)	3
11	Group 4	Small	9	Rural	Low (<1)	2
12	Group 4	Very large	5	Urban	High (2 to <5)	1

a HIV testing data was grouped using quartiles (Q1-Q3)^109^ which spilt the data into four equal-sized groups where Group 1 was the lowest value and Group 4 was the highest value for testing rates. NB The values were tests per 1000 population for 2022.

b Small: < 10 000; medium: 10 000 – 20 000; large: 20 000–30 000; very large: >30 000.

c 1 = most deprived and 10 = most affluent. IMD = Indices of Multiple Deprivation.

**Table 2.** Demographic characteristics – HCP interviews

Characteristics of HCPs (*N* = 25)	n
**Sex**	
Male	8
Female	17
**Ethnic group**	
White British	19
Asian British	4
Mixed	1
Not reported	1
**Experience, years**	
1≥5	11
6≥10	3
11≥20	6
>21	5
**Occupation**	
GP	20
Practice nurse	4
Pharmacist	1

HCP = healthcare professional.

### Scoping review findings

Knowledge of HIV testing guidelines and prevalence in the practice population is low among HCPs.^39,81–83^ HCPs also lack confidence and experience discomfort discussing HIV with patients and offering routine HIV tests.^16,81,82,100^ Concerns about patient reactions and stigma act as a barrier to initiating conversations about HIV.^82,84^ There was a lack of HCP awareness of the HIV stigma experienced by some minoritised groups.^81^

HCPs view routine HIV testing as time consuming because of the belief that pre- and post-test counselling and a lengthy consent process is required.^81,85,100^ HIV tests are not considered or included routinely when blood tests are requested^81^ and HCPs do not (typically) have a systematic way of recording behaviours that increase the risk of exposure to HIV so may not always recognise opportunities for testing.^82,86^

HCPs may also not recognise or be familiar with indicator conditions – and may not think about requesting an HIV test – particularly among those not considered typically ‘high risk’.^16,17,81,87,88^

Many of the PrEP reviews were conducted in the USA where the medication has been available longer than in England and where any HCP licensed to prescribe medication can prescribe PrEP. Despite this availability barriers to prescribing persist among primary care HCPs in the USA.

HCPs knowledge of PrEP is low.^53–65^ Most HCPs do not discuss PrEP with patients because of this lack of knowledge – as well as viewing PrEP as something that should be delivered by SHS.^51,54,56,60,61,65–68^

USA primary care HCPs report a lack of skills, knowledge, and time to counsel patients, to prescribe and carry out PrEP follow-up.^57,58,61,64,65^

HCPs experience discomfort discussing sexual activities/history with patients^54,57,59^ and have concerns about PrEP use leading to risky behaviour.^57,58,61,64,66,69^

### Interview findings

#### Capability barriers

Capability barriers centred around knowledge and awareness. Most HCPs were unaware of the local prevalence of HIV and/or national testing guidelines with those in high-prevalence-area practices indicating that their practice did not carry out routine testing as per the guidelines. Participants reported that testing was carried out in response to indicator conditions (for example, unexplained weight loss, lymphadenopathy) or risk factors indicating the need for an HIV test such as, intravenous drug use.

HCPs felt that a lack of skills and confidence in having a conversation with patients about HIV specifically and sexual health in general was a barrier to testing for some staff although many did not feel they lacked these skills themselves. A lack of knowledge and confidence among HCPs was also a barrier to discussing PrEP with patients. Most HCPs acknowledged that they knew very little about PrEP and few had discussed it with a patient.

HCPs expressed uncertainty about who might benefit from PrEP and felt that patients were better served by attending specialist services. Similarly, some participants felt that patients would know where to seek help:

*'I almost feel like people often know where to go for it and they know about it if they're in a high-risk group*.*'* (Participant 21, GP, high-prevalence area)

Indicator conditions were not known by all participants, and there was an acknowledgement that an inability to identify those who should be tested can lead to late HIV diagnosis:

*‘GPs often can miss those potential symptoms as indicative of HIV infection. I think they always tend to be thinking, it’s probably got to be something else, and it might be the fifth or the sixth thing they consider, rather than the first or the second.*’ (Participant 26, interest holder, low-prevalence area)

Despite this, there was a reported lack of systematic approaches to identifying individuals at increased risk of HIV.

There was a lack of standardisation across practices regarding tests offered during sexually transmitted infection (STI) checks. Some HCPs understood the need to include HIV/blood-borne virus (BBV) tests, but many preferred to refer patients to SHS:

*‘If they were requesting a routine STI screen, we would do the swabs here, but for blood borne viruses, we would generally refer to* [SHS]*.*’ (Participant 6, GP, high-prevalence area)

HCPs felt that SHS would provide a more streamlined and prompt service than general practice.

*‘It’s going to be much more efficient than us … and you’ve got the follow-up already organised.*’ (Participant 25, GP, low-prevalence area)

#### Capability facilitators

Facilitators for offering an HIV test include having knowledge and training about HIV, an interest in sexual health, experience of working in SHS, and being aware that patients face barriers accessing SHS:

*‘I know that for everybody I tell to go to* [SHS] *some of them won't go so I think “let’s just do it”'* [test for HIV]*.’* (Participant 16, GP, high-prevalence area)

HCPs suggested that HIV testing should be embedded in existing systems. For example, there was a view by many that HIV testing could and should be part of STI screening or that a well-designed screen prompt (pop-up) on the electronic patient record for indicator conditions could increase HIV testing:

*‘If you put in a code that … that could generate a prompt that says something like, have you considered a HIV test in this person, I think that’s – for me, that’s the only way.*’ (Participant 7, GP, low-prevalence area)

Several participants felt that training for HCPs would facilitate discussions with patients about PrEP:

*‘With the right training I would be very happy to.*’ (Participant 15, GP, low-prevalence area)

#### Opportunity barriers

Opportunity barriers related to limitations in staff time. Many participants discussed lack of time to carry out a test, often based on assumptions that a lengthy consent process was necessary. There were concerns that in a short consultation there was insufficient time to discuss the test and address patient questions:

*‘Time constraints wise is also the other one ... you could end up with a whole load of questions which perhaps you don’t have the time to answer in a sort of full way or knowledgeable way.*’ (Participant 20, nurse, low-prevalence area)

Participants in high-prevalence areas felt that testing according to guidelines would be challenging given the numbers involved. Most participants also felt that there was a lack of time in general practice to initiate, prescribe, or maintain PrEP prescriptions.

#### Opportunity facilitators

Staff suggestions for overcoming opportunity barriers to testing commonly focused on simplifying processes and ensuring that staff are aware that a lengthy consent process is not necessary for HIV tests.

Suggestions for normalising the process, for example, through integrating HIV tests with other blood tests or health checks was mentioned:

*‘I wish it was more normalised, for example maybe part of the NHS health check, you know, something very routine, but it almost ought to be added to that really, shouldn’t it?*’ (Participant 24, GP, low-prevalence area)

Although point-of-care tests were discussed as an option for increasing testing uptake, the perceived disadvantages of rapid tests (including accuracy and difficulties with finger prick tests) meant that this was rejected as a potential solution.

Conversations about PrEP could be prompted by consultations about sexual health, STIs, or by an HIV test.

#### Motivation barriers

Motivation barriers were linked to a reluctance to initiate conversations about HIV. There were several factors involved in reluctance to initiate conversations about HIV. Resources in general practice are stretched and participants described the competing priorities they face. They readily admit that preventive medicine (and HIV prevention) is not a priority:

*‘It’s not a priority … just getting through the day of clinical demand for things/symptoms that people have is the priority*.’ (Participant 14, GP, high-prevalence area)

Some HCPs felt that HIV stigma had an impact on the willingness to discuss testing with patients:

*‘I think there’s still a taboo about it … I don’t have a problem myself asking patients but yeah, I would guess some of my colleagues would certainly find it uncomfortable*.’ (Participant 15, GP, low-prevalence area)

HIV stigma was recognised as a barrier in small practices where patients might feel uncomfortable interacting with either a member of staff they may have known over many years or one that they may have a personal connection with:

*‘Most of our practice nurses and GPs have been here for quite a long time … So, the patient was more comfortable speaking to somebody that wasn’t within the practice.*’ (Participant 5, nurse, low-prevalence area)

Not all staff in smaller practices recognised this as a potential problem though. A few HCPs discussed the stigma faced by some patients in their communities if it was known they had had an HIV test. Some HCPs were aware of these barriers to accessing HIV tests and felt that they were hard to address. There may also be an impact on the motivation to discuss PrEP because of the consideration of stigma around HIV.

#### Motivation facilitators

Some HCPs were keen to follow testing guidelines if resources or financial incentives could be made available to carry out the additional tests:

*‘Without an enhanced service, so money* [for] *things and a budget it doesn’t happen.*’ (Participant 17, GP, high-prevalence area)

HCP and interest holders suggested that normalising the testing process would reduce stigma. For example, if it was a routine activity or part of another routine test so that staff could say to patients:

*‘... this is what we do for everyone.’* (Participant 25, GP, low-prevalence area)

A few HCPs mentioned that with appropriate funding they would be interested in initiating PrEP:

*‘In terms of PrEP I think it has to be some kind of service where we are funded to provide that.*’ (Participant 6, GP, high-prevalence area)

However, a lack of system-level commissioning and fragmentation of responsibilities for HIV services were highlighted by interest holder participants who considered that the distribution of funding was a key barrier to increasing general practice activity in HIV prevention:

*‘NHS England fund the treatment of HIV or commission it. The local authorities are meant to be doing the prevention … that’s all moving, so the treatment element of HIV is now being delegated to ICBs* [Integrated Care Boards] *… but it’s ... been done and still is being done* [on] *a regional type basis … we’re not commissioning as a system.’* (Participant 27, interest holder, high-prevalence area)

### Intervention development and refinement

#### Guiding principles

The intervention’s guiding principles (presented in Supplementary Table S2) centre on addressing low HCP motivation to test because of competing priorities and low awareness of HIV prevalence, testing guidelines and PrEP; time pressures to receive and deliver interventions; low self-efficacy to test and discuss PrEP; and the perception that SHS may be more appropriate settings for HIV initiatives.

Intervention components with relevant published evidence are as follows (Supplementary Table S3):

- Provide short impactful education to address lack of knowledge, skills and confidence around HIV testing and PrEP. To include: training to address lack of clinician knowledge and clinician anxiety associated with discussing HIV testing with patients;^16^ guidelines relevant to the HIV prevalence of the practice area, HIV epidemiology and indicator conditions;^82^ a modified version of a previously tested education intervention;^37^ education and training to address lack of clinician knowledge of PrEP and who may benefit from it, and lack of comfort discussing PrEP with patients^56,57^ and to persuade HCPs of the benefits of HIV testing and improving access to PrEP (given barriers to accessing SHS). The PrEP training content will also support general practice HCPs to identify opportunities to discuss PrEP with patients including but not limited to when testing for HIV.
- Introduce systematic ways of identifying when to test according to high- and low-prevalence areas. To include the provision of the BBV Test Alert (a prompt to test in response to indicator conditions).^17^ Electronic health record reminders have been effective in increasing HIV testing.^81,82,89^ Decision aids may improve motivation and capability.^90^ Using a standardised approach to HIV testing helps to alleviate discomfort^81^ and indicator condition testing programmes have been used effectively to increase HIV testing in primary care.^16^
- Simplify the approach to HIV testing. To address time barriers, simplify processes of consent,^81^ normalise HIV testing using opt-out approaches (without pre-test counselling) that are acceptable to the public^101^ and are proven to increase testing,^88,91^ and may reduce stigma.^81^
- Communicate HIV policy to patients to raise awareness about the practice’s approach to HIV testing and raise awareness of PrEP and who may benefit from using it and normalise testing processes through regularly communicated information on HIV.
- Provide leadership and support to implement the intervention through practice champions shown to raise awareness, support and educate colleagues about initiatives to increase HIV testing, and to develop ideas and promote shared learning.^84^
- Systematise the signposting of eligible patients to PrEP services.

#### Intervention – co-production and optimisation

Four focus groups comprising 16 HCPs from eight general practices and three focus groups involving 13 members of the public were carried out between November 2023 and April 2024. Table 3 provides demographic information.

**Table 3.** Demographic characteristics of focus groups

Group and characteristic	*n* ^a^
**HCP participants (*N* = 16)**	
Sex	
Male	8
Female	8
Ethnicity	
White British	13
White Other	1
Asian British	1
Black African	1
Experience, years	
1≥5	4
6≥10	6
11≥20	4
>21	2
Occupation	
GP	14
Practice nurse	1
Nurse associate	1
**Public participants (*N* = 13)**	
Gender	
Male	3
Female	10
Ethnic group	
White British	4
Black African	5
Other	1
Not reported	3
Experience of HIV test or PrEP	
Yes	4
None	4
Not reported	5
Age, years	
Range	19–42
Average	29
Not reported	3

^a^Data are *n* unless otherwise indicated. HCP = healthcare professional. PrEP = pre-exposure prophylaxis.

#### HCP focus group feedback

HCPs viewed many aspects of the intervention positively, for example, simplifying the consent and counselling process. HCPs emphasised the need for training to be brief and in video format to ensure ease of access:

*‘I think, you know, I guess the aim if you could keep it short, succinct, 15/30 minutes then you can give people time for that. But if it starts to get longer than that, you know, that becomes quite a lot of pressure, especially if all of the clinicians need to do it. You know, that would mount up quite a lot.*’ (HCP focus group 4, Participant 1, GP)

Simplifying test processes was key:

*‘It’s probably much easier to add it on to other tests that we’re doing rather than getting every single new patient to come in for a blood test.*’ (HCP focus group 1, Participant 1, GP)

Also key was the need to normalise HIV testing:

*‘If you see someone with thrush a few times then you think, you know, maybe I should do a blood sugar* [test]*. I think we all routinely think about that now but maybe 30 years ago we didn’t and I suspect this will happen with HIV, that it will become a bit more standardised.*’ (HCP focus group 2, Participant 2, GP)

The importance of informing all patients about the approach to patient consent using opt-out principles was emphasised:

*‘So I think it would be good to have that openness with the patients and always tell them that yes we want to do this test but you have got the uhm … the ball is in your court so you can decide whether you want to opt-out or not rather than for them to find out that we* [have] *done it without their knowledge cos I think once the trust is gone it will be gone.*’ (HCP focus group 3, Participant 2, nurse)

There were some concerns expressed about the potential for patient confusion about an opt-out approach (that is, not knowing whether an HIV test had been done), particularly non-English speaking patients or those without access to GP digital communication systems. HCPs emphasised the need to ensure that non-English speaking patients received appropriate communication in their own language.

Some expressed concerns about pop-up fatigue regarding the BBV Test Alert:^17^

*‘I think often, with pop-ups though, if they're coming up on every – you know, on loads and loads of patients, you do just tend to switch off.*’ (HCP focus group 1, Participant 1, GP)

However, many felt that the advantages of a system that aids decision making would outweigh this:

*‘I think it’s probably a good reminder having it that way, and especially having an easy-to-use kind of template as well with clear tick boxes and counselling advice.*’ (HCP focus group 2, Participant 1, GP)

Some felt that these systems would help to normalise HIV and hence reduce stigma.

#### Public focus group feedback

Public feedback was largely positive with some caveats. Participants focused on the importance of communicating the approach to HIV testing effectively:

*‘I would like it verbally mentioned and I’d probably be like yeah, yeah, that’s fine, but it needs to be clear.*’ (Public focus group 1, Participant 1)

*‘I also find the television, the screen in the GP very useful for sending information because while we are sitting in the waiting room, that's* [...] *the place that we all look.*’ (Public focus group 3, Participant 5)

Participants also suggested wording that would indicate that testing was universal so that patients would not feel targeted.

Public participants said that it was important that patients were aware that they were being tested and are informed that they can opt-out. One participant commented on the convenience of the opt-out process:

*‘I think it sounds like a really good idea, it would be really convenient for me.*’ (Public focus group 2, Participant 1)

Participants felt that communication information should be simple, engaging and include texting, posters, and practice screens. Public participants also emphasised the importance of communicating with non-English speaking people.

The convenience of including an HIV test as part of STI screening was considered key:

*‘If you’ve already taken the blood test, you may as well just go for it because at that point we’re already in. It’s better than having to come back and do another test and keep on doing it. The blood’s coming out anyway.*’ (Public focus group 1, Participant 1)

Participants wanted access to HIV tests and PrEP to be available at general practices (rather than travelling to sexual health clinics) and this was particularly important for those living in rural communities. Discussing access to PrEP through GP practice one focus group participant stated:

*‘Sexual health clinics is an extra layer of like okay, I’ve got to find this different special clinic and I’ve got to find a different set of contact details and work out how the system works and where they are, it’s like it’s a lot more hassle* […] *Like it makes sense for sexual health clinics to offer it but it would make sense for them both to offer it.'* (Public focus group 2, Participant 1)

These participants felt that GPs should have the knowledge and training to be able to communicate to patients about PrEP.

**Figure 2.**
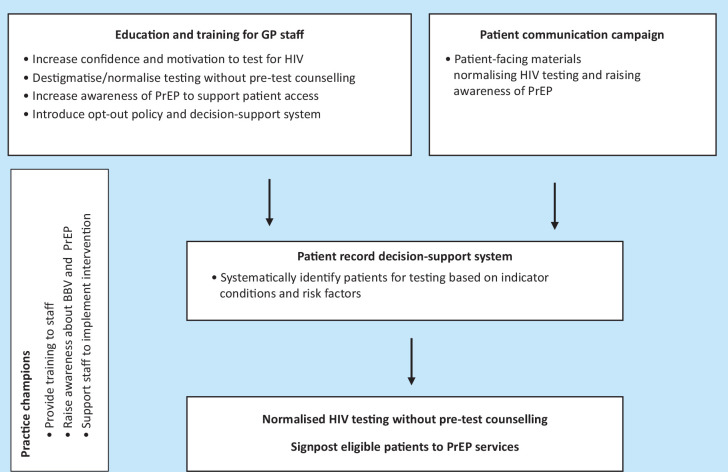
Intervention optimisation – key design features to increase HIV testing and access to PrEP. BBV = blood-borne virus. PrEP = pre-exposure prophylaxis.

Figure 2 shows the key design features of the multifaceted intervention to increase HIV testing and access to PrEP.

## Discussion

### Summary

This paper outlines the development of a multifaceted intervention to support HIV testing and access to PrEP in general practice using PBA. In summary the proposed intervention mapped to elements of the COM-B model involves:

- HCP education and training tailored to the HIV prevalence of the practice population to raise awareness of HIV testing and PrEP;
- a streamlined approach that normalises testing and integrates the process into routine care;
- a clinical decision-support application embedded within the electronic health record;
- a patient communication campaign; and
- the introduction of practice champions to support the intervention implementation.

Research is now needed to explore the feasibility and the effectiveness of the intervention. If efficacy can be demonstrated, this intervention has the potential to support the goal to eliminate new cases of patients with HIV by 2030.

### Strengths and limitations

A strength of this research is the use of a codesign approach to develop materials, integrating insights from academic literature, qualitative interviews, behavioural science theory, and patient and public involvement. This allowed us to gain a comprehensive understanding of the context and differing perspectives, identify a wide range of barriers to testing and supporting access to PrEP, and collaboratively discuss potentially accessible, acceptable, persuasive, and feasible approaches to address them. We aimed to be as inclusive as possible and used various approaches to collect data from a range of practices, locations, HCPs, and members of the public. Such widescale involvement may help optimise the acceptability, accessibility, and feasibility of the intervention.

Despite best efforts, key voices may have been missed including people who do not read, write, or speak in English. Given the need for communication with the public about opt-out testing, it is critical that we engage with the latter before the intervention is implemented.

### Comparison with existing literature

In line with previous research^81,102^ we found testing behaviour to be influenced by HCP’s capability, motivation, and opportunity to offer tests. Indeed, a key theme from the planning stage highlights that most HCPs are not aware of the local HIV prevalence, and did not know what this meant in terms of the HIV testing guidelines.^39,81–83^

To increase testing in accordance with guidelines, it was clear that there is a need to raise awareness of the testing guidelines and HIV prevalence in the local area. The findings highlighted how educational materials should be delivered to work within general practice (for example, must be short, online, and be delivered flexibly). Intervention content targeting knowledge also needed to go beyond the provision of information and must also include prompts or reminders when patients present with indicator conditions.^88^

A few HCPs interviewed in the study identified pop-ups as a way to facilitate testing according to indicator conditions and in focus groups HCPs found this acceptable to increase the identification of people living with HIV and reduce late diagnosis. Currently, testing by indicator conditions is unevenly and insufficiently implemented across conditions and healthcare settings.^88,103^ However, interventions using indicator conditions to guide testing have increased testing rates and diagnoses.^16,87^ For example, implementation of a normative illness script-based decision-making model, called BBV Test Alert, prompts HCPs to add a BBV test in response to indicator conditions. A pilot of the Test Alert led to five times more HIV testing and three times more hepatitis B and hepatitis C testing although rates subsequently decreased. Although the pilot demonstrated the Test Alert’s feasibility, research is needed to determine its effectiveness and cost-effectiveness.^17^ Testing returned to baseline levels (declining from month 2 to month 6) potentially because of alert fatigue or because they had reached the population requiring testing.^17^ However, the model also increased consultation number and time,^17^ thus this approach in isolation may not be appealing to HCPs.

In addition to barriers relating to knowledge of HIV and PrEP, motivation and capacity for testing was also low. For educational campaigns to be effective they needed to be paired with approaches for simplifying the testing process. Indeed, many HCPs were reluctant to offer a test or initiate conversations about PrEP because of a lack of time. This was in part driven by the perception that it would be necessary to engage in lengthy conversation and pre-test counselling with the patient before a test could be offered. Many HCPs assumed that patients would have concerns and questions if they were to discuss HIV. Patients who may benefit from PrEP were also assumed by HCPs to know where they could access it, whereas data on PrEP access suggests there is a need to improve awareness of PrEP among key groups^104^ and barriers to access through SHS exist.^6^

Motivation was reduced when HIV was perceived as a low priority, as even in high-prevalence areas, 2 per 1000 practice population was considered a small number by our participants. Although numerous approaches to increasing motivation and reducing stigma were identified, the most acceptable approach needed to reduce rather than extend consultation time. Opt-out approaches to patient consent to testing^105^ — in which a person is informed that HIV testing is routine/standard of care and they actively decline if they do not wish to be tested for HIV — may help address these barriers to testing and may help reduce the time to discuss testing, reduce stigma,^81^ and normalise testing.^105^ Opt-out approaches have been successfully adopted within antenatal services and showed promise for new patient registrations in an area with high HIV prevalence.^26^ BBV opt-out testing in emergency departments within very-high-prevalence areas has led to increased testing in England.^106^ Using a combination of previous successful strategies including indicator condition-guided testing, training and education, and opt-out approaches could be a feasible and acceptable approach to increase HIV testing across areas of low and high HIV prevalence.^88^

Structural barriers noted by interest holder participants (for example, the fragmentation of funding responsibilities for HIV services) have made it challenging to deliver coordinated and planned care across the system to meet people’s needs.^107^ This has led to disparities in types of HIV tests and testing opportunities across the system.^108^ New BASHH/BHIVA guidance recommends expanding PrEP provision beyond SHS including primary care (with regulation from SHS)^31^ and there are plans to explore piloting HIV PrEP referrals from primary care to specialist SHS.^104^ General practice interventions to support access to PrEP are therefore timely.

### Implications for research and practice

To change HIV testing practice, our results suggest we would need to address:

- capability barriers including a lack of knowledge and awareness of who, when, and how to test;
- opportunity barriers including reducing the amount of time it takes to offer and deliver a test; and
- motivational barriers, for example, offering financial incentives.

This research has led to the development of an intervention that is potentially acceptable to HCPs and patients, however, research is needed to test the real-world feasibility and effectiveness of the intervention for increasing HIV testing and supporting access to PrEP. Although there is promising evidence for each aspect of our co-produced intervention in isolation, there is a need for evaluation of the intervention as a whole. However, the wider context of structural fragmentation of responsibility for HIV will also need to be addressed to facilitate increased HIV testing and access to PrEP.

## Data

Data will be available at the University of Bristol data repository, data.bris. Data access will be restricted to bona fide researchers for ethically approved research and subject to approval by the University’s Data Access Committee.
